# Heavy episodic drinking and soccer practice among high school students in Brazil: the contextual aspects of this relationship

**DOI:** 10.1186/1471-2458-13-247

**Published:** 2013-03-20

**Authors:** André Bedendo, Emérita S Opaleye, André Luiz Monezi Andrade, Ana Regina Noto

**Affiliations:** 1 Departamento de Psicobiologia, Universidade Federal de São Paulo UNIFESP, Rua Botucatu, 862 1º andar Ed. Ciências Médicas, CEP 04023–062, São Paulo, Brasil

**Keywords:** Soccer, Team sports, Binge, Alcohol, Students, Culture

## Abstract

**Background:**

Heavy episodic drinking (HED) (consumption of five or more drinks on the same occasion) among adolescents is related to several problems and partaking in sport or physical activities has been suggested as an option to prevent or reduce alcohol consumption among this population. The aim of this study was to investigate the relationship between soccer practice and heavy episodic drinking among high school students from Brazil.

**Methods:**

Data were obtained from a cross-sectional study among a representative sample of public and private high school students from all Brazilian state capitals (N=19,132). Only students aged from 14 to 18 who reported having taken part in soccer practice, other team sports or non-practicing sports in the last month were included. Characteristics of sport practice (frequency and motivation) and HED in the last month (type of drink; where and with whom they drank; frequency of HED) were also considered. Regression models were controlled for sociodemographic variables.

**Results:**

For all groups studied most of the students reported drinking beer, with friends and at nightclubs or bars. Soccer practice was associated to HED when compared to non-practicing sports and to other team sports. Compared to other team sports, playing soccer for pleasure or profession, but not for keep fit or health reasons, were more associated to HED. Frequency of soccer practice from 1 to 5 days per month and 20 or more days per month, but not from 6 to 19 days per month, were also more associated to HED.

**Conclusions:**

The relationship between soccer and HED appears to be particularly stronger than in other team sports among adolescents in Brazil. Induced sociability of team sports practice cannot be assumed as the main reason for HED among soccer players. Possibly these results reflect the importance of a strong cultural association between soccer and beer in Brazil and these findings should be integrated to future prevention or intervention programs.

## Background

Heavy episodic drinking (HED) or binge drinking (consumption of five or more drinks on the same occasion) among adolescents is related to several problems such as impaired memory, academic issues, violence, unprotected sex, getting hurt or injured, damaging properties and dangerous driving [1-3]. In spite of this, data from the 2009 National Youth Risk Behavior Survey indicated that 60.9% of United States high school students who are current drinkers reported HED in the last month [4]. In Europe a study showed that 43% of adolescents reported HED in the last month [5]. In Brazil, approximately one third of students from private schools of São Paulo reported this pattern of use [6].

Some studies have suggested the importance of sports or physical activities in intervention programs in order to prevent or reduce alcohol consumption among high school students [7,8]. In contrast, other authors have indicated that high school students engaged in sports or physical activities are more likely to report alcohol use, including HED [9-11]. Furthermore, adolescents playing team sports are more likely to report alcohol use and intoxication than those playing individual sports [10,11].

Another sport characteristic suggested as a differentiating factor for alcohol use is the frequency of sport practice [11,12]. Peretti-Watel and colleagues [13] observed that high school students who practiced sports at a higher frequency reported at least one episode of drunkenness in the last month compared to students with a lower frequency of practice. However results are still insufficient for a conclusion about which frequencies are more related with a lower or higher level of alcohol consumption. In addition, both motivation for sports participation and for drug use were also suggested as important parameters in the relationship between sports and drug use [14]. However most of the studies explored only the motivation for the substance consumption, not for the sport practice [15,16].

Prior studies pointed out the importance of social aspects of sports practice on drug use among adolescents [17,18]. In this sense, some studies used Bronfenbrenner’s Ecological Systems Theory, involving the multiple levels of the surrounding milieu [19,20] as theoretical grounding for studying substance abuse among adolescents [21,22]. Brazilian culture is strongly associated to soccer and its practice is wide spread, which has led to Brazil being called “*The country of soccer*”. Moreover, soccer is one of most practiced sports in Brazilian high school physical education classes [23] and is an important opportunity for social interaction among adolescents. On the other hand, alcoholic beverages are commonly associated with sports in some countries. A prior study suggested that the relationship between sports and alcohol seems to be particularly strong to soccer (or football, as called in other countries) [24]. For example, in The United States, beer advertising is culturally associated with baseball and American football, in Brazil, it is linked to soccer [25].

Further research exploring the involvement of the population with the practice is necessary to expand the understanding about the relationship between sports and alcohol consumption, especially in developing nations [9,26]. This knowledge is important to design preventive programs for adolescents, which take into account the contextual diversity [27]. This study aims to explore the relationship between HED and contextual factors of soccer practice among high school students, with a representative sample from all the 27 state capitals of Brazil, the largest country of Latin America. Taking into account the fact that Brazilian culture is strongly associated to soccer and that this sport is related to alcohol consumption, especially when practiced in a pleasure context, our hypothesis is that soccer practice is particularly associated to HED. Furthermore, this relationship also should be influenced by frequency of and motivation for practice.

## Methods

### Participants

Our study is part of major cross-sectional survey conducted by the Brazilian Psychotropic Drug Information Center (Centro Brasileiro de Informações sobre Drogas Psicotrópicas - CEBRID) with a representative sample of students from all Brazilian state capitals. Data were collected from March to October 2010. Using a national governmental register of all educational institutions, we obtained a list of all private and public schools for each one of the 27 cities and we created independent subsamples of public and private schools. For each subsample, three stratums were considered: elementary schools, high schools, and schools with both levels. For each one of them we allocated a proportional sample according to the number of students (estimated by the number of classrooms in each school). The process generated a final sample size of 50,890 students attending 789 schools (512 public and 277 private).

Our analyses were restricted to high school students (24,280). A response rate of approximately 80% was obtained (with a 20.5% absence rate on the day of the survey and 0.3%f refusal rate) totaling 19,230 questionnaires. In order to reduce over-reporting, a question with a fictitious drug was included and students that positively answered this question were excluded (n=98). Students who were not aged from 14 to 18 were also excluded (n= 1,835); as well as those who practiced non-team sports (n=3,659) and those with missing data to any variables (n=3,752). Thus, the final sample considered in this study was from 9,886.

### Procedures

Self-report questionnaires were completed in the classroom and supervised by a group of trained interviewers. The training covered issues related to the attitude of the interviewer, ethics and procedures related to questions or concerns the students might have.

The complete application procedure lasted for about 50 minutes, after that, the completed questionnaires were sealed in front of all the students. In order to ensure the confidentiality of the application, none of school staff were able to be present during this stage. The visit to every school was performed in a single day to avoid possible contamination. All participants were informed about their voluntary participation and the aims of the survey. Furthermore the students were notified that at any time they could withdraw their participation and that all data would be used for research purposes only. The study was approved by the Research Ethics Committee of the Universidade Federal de São Paulo (process CEP 0348/08).

### Measures

The questionnaire was based on a World Health Organization instrument [28] and European School Survey Project on Alcohol and Other Drugs (ESPAD) questionnaire [5]. These questionnaires are specially designed for self-report surveys about substance use among students.

Sociodemographic variables: were obtained by questions regarding age, gender and type of school (public or private).

Sports variables: data were obtained regarding the previous 30 days and were accessed by questions about the sport most frequently practiced (non-players; gym or bodybuilding; athletics, cycling or swimming; basketball, volleyball or handball; dancing or gymnastics; soccer; fighting), the frequency of sport practice (1 to 5 days/month; 6 to 19 days/month; 20 or more days/month) and motivation for the sport (pleasure; keep fit or health reasons; profession). For the team sports subsample, only those reporting basketball, volleyball or handball players were considered.

Heavy Episodic Drinking: it was assessed by the question “In the last month how many times have you had 5 or more drinks at the same time”. The answers were pre-codified as none; 1–2 times/month; 3–5 times/month and 6 or more times/month. The questionnaire included an explanatory table with the equivalence of doses (approximately 14 grams of alcohol/drink). For some analyses, the responses were grouped into a dichotomous variable (0 – no; 1 – yes). This drinking pattern was also discriminated by beverage type (beer; vodka; other beverages - cachaça, whisky, brandy, caipirinha, ice, liquor, champagne or wine), preferential places that the students drank *nightclubs or bars; friends’ houses; at home or in relatives’ houses* and with whom students drank *alone; relatives; friends*. Type of beverage, places of drinking and with whom students drank were multiple answer questions.

### Data analysis

Population characteristics regarding sociodemographic variables and HED of non-players, team sports and soccer players were described. Two logistic regression models were used to estimate Odds Ratios (OR) for HED. The first logistic regression model (LRM1) was the crude model and the second model (LRM2) was adjusted for sociodemographic variables. Sports (non-players, team sports or soccer), frequency or motivation for the practice were considered as independent variables. All analyses were performed considering the sample weights and using STATA version 11. The minimum level of significance adopted was 5%.

## Results

### Sample characteristics

Characteristics of non-players, team sports and soccer players are described in Table 1. Regarding the total sample, the majority of students were at public school (81.8%, 95% CI: 78.8-84.5), were female (57.3%, 95% CI: 55.9-58.6) and the mean age was 15.9 years (Standard Error=0.03). Among those who played soccer or team sports, they commonly did this from 1 to 5 days per month and for pleasure.

**Table 1:** Sample characteristics and associations between non-players, team sports and soccer players and potential confounders (N= 9,886)

		**Non-players(n= 4,783) wgt% (95% CI)**	**Team Sports**^**a **^**(n= 1,788) wgt% (95% CI)**	**Soccer(n= 3,315) wgt% (95% CI)**	***p value***
	**n (total)**				
***Age (years)***					
14	1016	8.0 (6.6–9.6)	14.3 (11.1–18.2)	11.1 (8.9–13.7)	<0,001
15	2753	25.5 (22.7–28.4)	30.4 (26.3–35.0)	29.9 (26.8–33.1)	
16	3028	30.1 (27.3–33.0)	31.1 (27.0–35.5)	29.3 (26.4–32.3)	
17	2275	26.4 (23.9–29.2)	18.6 (15.5–22.2)	20.9 (18.4–23.6)	
18	814	10.0 (8.6–11.6)	5.5 (4.2–7.2)	8.9 (7.3–10.8)	
***Gender***					
*Female*	5687	79.0 (77.2–80.7)	73.1 (69.5–76.4)	18.4 (16.3–20.7)	<0,001
*Male*	4177	21.0 (19.3–22.8)	26.9 (23.6–30.5)	81.6 (79.3–83.7)	
***Type of school***					
*Public*	6347	84.5 (81.6–87.0)	75.0 (69.3–79.8)	81.5 (77.8–84.7)	<0,001
*Private*	3539	15.5 (13.0–18.4)	25.1 (20.2–30.7)	18.5 (15.3–22.2)	
***Frequency of practice (per month)***					
*1 to 5 days*	2614	*----*	65.6 (62.5–68.7)	45.8 (43.0–48.6)	<0,001
*6 to 19 days*	1284	*----*	21.4 (18.7–24.4)	25.2 (22.9–27.6)	
*20 or more days*	1103	*----*	12.9 (10.6–15.7)	29.0 (26.7–31.4)	
***Motivation for practice***					
*Pleasure*	2715	*----*	80.9 (76.8–84.4)	76.3 (73.4–79.0)	<0,001
*Keep fit or health reasons*	558	*----*	15.6 (12.8–18.8)	14.7 (12.7–17.0)	
*Profession*	214	*----*	3.5 (2.0–6.1)	9.0 (6.8–11.7)	
***Heavy episodic drinking last month***					
*No*	8232	83.3 (81.1–85.3)	86.1 (82.9–88.8)	79.1 (76.5–81.4)	<0,001
*Yes*	1654	16.7 (14.7–18.8)	13.9 (11.2–17.1)	21.0 (18.6–23.6)	
***Frequency of heavy episodic drinking last month***					
*1 to 2 times*	1008	60.0 (54.6–65.2)	71.8 (61.7–80.1)	54.8 (49.5–59.9)	0,041
*3 to 5 times*	404	24.7 (20.4–29.6)	17.3 (11.2–25.6)	25.9 (22.2–30.0)	
*6 or more times*	242	15.3 (12.2–19.2)	11.0 (6.3–18.4)	19.3 (14.8–24.8)	
***Type of drink***^**b**^					
*Beer*	2031	69.4 (65.7–72.8)	61.4 (55.1–67.4)	69.4 (65.3–73.3)	0,021
*Vodka*	456	11.4 (9.6–13.5)	17.3 (12.9–22.8)	15.4 (12.2–19.3)	
*Other beverages*	516	19.3 (16.3–22.6)	21.3 (16.2–27.5)	15.2 (11.9–19.1)	
***Where they drink***^**b**^					
*At home or relatives houses*	780	33.3 (29.4–37.5)	30.4 (24.2–37.3)	27.0 (22.7–31.7)	0,120
*Nightclub or bars*	1223	44.4 (40.2–48.6)	46.3 (39.3–53.5)	51.5 (47.2–55.8)	
*Friend’s houses*	646	22.3 (19.0–26.0)	23.3 (18.4–29.1)	21.6 (18.5–25.0)	
***With whom they drink***^**b**^					
*Alone*	100	2.9 (1.9–4.5)	3.3 (1.7–6.4)	4.3 (2.7–6.7)	0,006
*Relatives*	1257	46.7 (42.9–50.6)	46.9 (40.0–53.8)	37.1 (32.9–41.6)	
*Friends*	1604	50.4 (46.5–54.2)	49.8 (43.0–56.7)	58.6 (54.0–63.1)	

wgt - weighted.

^a^ Basketball, volleyball or handball.

^b^ Multiple answers question.

Table 1 also presents the characteristics of HED. Regarding the frequency of HED last month, soccer players reported a higher frequency of HED (6 or more times) than non-players and team sports players (19.3%, 95% CI: 14.8-24.8). The most popular beverage reported by all three groups of students was beer, with students usually drinking with friends and at nightclubs or bars.

### Correlates of HED

At least one HED in the last month was reported by 17.7% of students (95% CI: 16.0-19.4). We first conducted the two logistic regression models for HED comparing students who reported soccer practice in the last month and non-players. Soccer players were more associated to HED both in LRM1 (OR=1.3 95% CI: 1.1-1.6) and LRM2 (aOR=1.3 95% CI: 1.1-1.6) (Table 2). In order to compare soccer to other team sports practice, another two logistic regression models were conducted (Table 3). Soccer players were also more associated to HED than team sports practitioners in both models.

**Table 2:** Crude and adjusted odds ratios for heavy episodic drinking last month for soccer players compared to non-players (N=8,098)

		***Total***	**Crude OR(95% CI)**	**Adjusted OR**^**a**^**(95% CI)**
	**n**	**wgt% (95% CI)**		
***Sports***				
*Non-players*	4783	58.6 (56.7–60.5)	1	1
*Soccer*	3315	41.4 (39.5–43.3)	1.3 (1.1–1.6)*	1.3 (1.1–1.6)*
***Age (years) - mean (SE)***	8098	16.0 (0.03)	1.4 (1.3–1.5)*	1.4 (1.3–1.5)*
***Gender***				
*Female*	4431	53.9 (52.2–55.6)	1	1
*Male*	3648	46.1 (44.4–47.7)	1.3 (1.1–1.6)*	1.1 (0.9–1.4)
***Type of school***				
*Public*	5392	83.3 (80.3–85.9)	1	1
*Private*	2706	16.7 (14.1–19.7)	1.5 (1.2–1.9)*	1.7 (1.3–2.2)*

SE - Standard Error ; wgt - weighted.

^a^ Adjusted by age, gender and type of school.

* p<0,05.

**Table 3:** **Crude and adjusted odds ratios for heavy episodic drinking last month for general team**^**b **^**sports and soccer practice by frequency and motivation (N= 5,103)**

		***Total***	**Crude OR(95% CI)**	**Adjusted OR**^**a**^**(95% CI)**
	**n**	**wgt% (95% CI)**		
***Sports***				
*Team Sports*	1788	33.8 (30.8–37.0)	1	1
*Soccer*	3315	66.2 (63.0–69.3)	1.6 (1.3–2.1)*	1.6 (1.2–2.1)*
***Sports (by frequency per month)***				
***1 to 5 days***				
*Team Sports*	1097	42.4 (38.4–46.5)	1	1
*Soccer*	1517	57.6 (53.5–61.6)	1.7 (1.1–2.5)*	1.6 (1.0–2.4)*
***6 to 19 days***				
*Team Sports*	425	30.4 (26.2–35.1)	1	1
*Soccer*	859	69.6 (64.9–73.8)	1.4 (0.9–2.3)	1.4 (0.8–2.6)
***20 or more days***				
*Team Sports*	236	18.6 (15.2–22.7)	1	1
*Soccer*	867	81.4 (77.3–84.9)	2.0 (1.0–3.9)*	2.4 (1.2–5.1)*
***Sports (by motivation)***				
***Pleasure***				
*Team Sports*	930	34.7 (31.3–38.4)	1	1
*Soccer*	1785	65.3 (61.6–68.8)	1.9 (1.3–2.7)*	1.7 (1.2–2.6)*
***Keep fit or health reasons***				
*Team Sports*	215	34.7 (28.9–41.0)	1	1
*Soccer*	343	65.3 (59.0–71.1)	1.2 (0.6–2.3)	1.0 (0.5–2.0)
***Profession***				
*Team Sports*	39	16.4 (11.0–23.8)	1	1
*Soccer*	175	83.6 (76.2–89.0)	6.5 (1.4–29.2)*	4.6 (1.3–16.2)*

wgt - weighted.

^a^ Adjusted by age, gender and type of school.

^b^ Basketball, volleyball or handball.

* p<0,05.

Analyses comparing non-players to soccer players showed that those who have practiced this sport from 6 to 19 days or for 20 or more days per month were more associated with HED in all logistic regression models. In relation to motivation, LRM1 and LRM2 showed that practicing soccer for pleasure was more associated to HED (results not shown).

Once it was established that the frequencies of 6 to 19 days and 20 or more days per month and pleasure motivation of soccer practice (compared to non-players) were more associated with HED, we conducted new logistic regression models in order to compare soccer players to players from other team sports (Table 3). LRM2 showed that soccer players who practiced sports from 1 to 5 or 20 or more days per month reported more HED than team sports players with same frequency of practice (aOR=1.6 95% CI: 1.0-2.4 and aOR=2.4 95% CI: 1.2-5.1), respectively. Regarding the motivation, LRM2 has demonstrated that those soccer players who played for pleasure or those playing for profession also reported more HED than students playing other team sports with the same motivations. (aOR=1.7. 95% CI: 1.2-2.6 and aOR=4.6 95% CI: 1.3-16.2), respectively.

## Discussion

Our data indicates that playing soccer was more associated to HED in the previous month compared to non-playing sports. Compared to other team sports, soccer practice was also more related to HED. Yet compared to other team sports, soccer players with the lowest and highest frequencies of practice as well as those motivated for pleasure and for profession were more prone to report HED. This data confirmed our primary expectations of soccer players being associated to HED and that frequency and motivation for practice are differential factors when predicting HED. Regarding preferred beverages, drinking partners and places for drinking, subtle differences were observed in our study. All groups studied reported beer as the most preferred beverage, drinking mostly with friends and nightclubs or bars being the most common setting.

Soccer was more associated with HED in the last month when compared to non-practicing sports. Similar results were observed by previous studies that pointed to a high level of alcohol consumption among high school students who played sports or practiced physical activities [9-11]. The importance of social contexts of sports practice for drug use were highlighted recently by other another study [29]. Socialization aspects of adolescent development may exert some influence on the relationship between sports practice and alcohol use. Studies showed that peer norms have an important role in physical activity behavior among adolescents [30] and peer group pressure is a predictor of substance abuse [31]. Accordingly, it is expected that once alcohol consumption is a part of normative culture, the adolescent could behave in a similar manner to that of their peers in order to promote the social bonds. Possibly when practicing soccer, adolescents are involved with older peers, and being involved with older friends or acquaintances allows easier access to beverages from this social source [32], favoring alcohol consumption, especially because in Brazil the legal drinking age is 18. Therefore, future studies should compare team and individual sports in order to explore collectivity and peer roles as an influence on alcohol consumption related to sports practice.

A previous study indicated that the induced sociability could be a more important factor than the sport practiced in relation to alcohol use [13]. In team sport practice, the individual could be influenced by normative attitudes of the group. On the other hand, our data suggests that the practice of a team sport and its socialization aspects are not the only reason for higher HED reporting among adolescents. Soccer players were more likely to report HED than other team sports players. Soccer and beer are highly associated in Brazilian culture and this relationship apparently plays a fundamental role in alcohol consumption by adolescents. Even though partaking in sports is usually regarded as a healthy practice, there is evidence that culture exerts influence on sports players drug use, and its practice is often accompanied by alcohol use [33]. Thus, the possible socialization induced by team sport practice itself cannot be directly assumed as a unique factor associated with the risk of HED, it being important to consider other aspects as cultural issues of the sports practice.

In our data, playing soccer for pleasure compared to team sports was associated with HED. A previous study that recreational physical activity does not provide much protection in terms of alcohol use [34]. Pleasure, as a recreational motivation, is related to a lower commitment to the sport practice. Once the practitioner is not concerned about how much their performance will be impaired by alcohol consumption this could possibly promote drug use. On the other hand, adolescents who reported playing soccer as a profession also reported more HED than those with the same motivation and practicing other team sports. In contrast with those who play for pleasure, players with professional motivation for the practice tend to be more devoted to the practice. This motivation presumes a higher commitment to the sport. Therefore, these more athletic students could experience more stress and pressure associated with maintaining their dual activity, which could increase the risk for alcohol use [35].

Both highly active practitioners and those with the lowest frequency of practice of soccer were related to more HED. Independently of being highly or slightly active, soccer players were more prone to report HED. These results reinforce the idea of the strong cultural bond between soccer and alcohol. This connection seems to be so strong that regardless of the motivation (pleasure or as a profession) or frequency (1 to 5 days or 20 or more days per month), HED is more frequent among soccer than in other team sports.

In regard to HED characteristics, students independently of being non-players, soccer or team sports players reported beer as the most frequent beverage consumed. This beverage was previously reported as the predominant drink among adolescents in Brazil and several others countries [5,36]. The beverages preferences among youth could promote a better understanding of related factors and more effective interventions could be tailored [37]. Soccer players reported drinking more frequently than non-players or team sports students. Thus, they possibly could experience more the consequences of HED and be more likely to engage in risky behaviors.

Our results also indicated that all three student groups usually drink with their friends and at nightclubs or bars. It is also interesting to note that the adolescents also frequently drink with their relatives. Several studies pointed out the influence of parents and relatives behaviors and practices on adolescents drinking behaviors [38-40], including parents drinking behaviors [41]. As parents and relatives are generally the most involved with the adolescents, their approval on drinking behavior may be important in explaining their alcohol use [40].

Several other authors have suggested sports-based interventions programs for specific groups, including high school students [7,8,42,43]. Our study results suggest the need to consider the associated aspects of sports practice such as motivation, frequency and especially the culture, for intervention programs among this population. A recent study showed that sports clubs using accredited programs to reduce alcohol risk have a reduction in short and long-term risky drinking among members whereas clubs not using such programs have higher levels of alcohol consumption [44]. This data suggests that tailored programs could be useful for reducing alcohol use, and our data could be integrated to improve future adolescent programs which aim to reduce alcohol consumption.

### Strengths and limitations

This is the first study to assess the relationship between soccer and heavy episodic drinking among a representative sample of high school students from public and private schools of Brazilian state capitals. However, the results presented in this paper should be considered taking into account its strengths and limitations. This study is related to adolescents who were at high schools. Thus, our data should not be extrapolated to 14,7% of adolescents aged 15 and 17 that were not at school [45]. In addition, our sample is representative from the 27 Brazilian state capitals, and adolescents from rural areas or country towns were not assessed.

Students might under-report their own substance use and students absent on the day of the survey could also have different patterns of alcohol use or sport practice. Furthermore, the data should be not directly extrapolated to other countries, especially those in which soccer is not a primary sport practiced. Notwithstanding, this study could serve as an example for other countries with a strong cultural relationship between sports practices and drugs use.

When multiple statistical comparisons are carried out, as in this paper, the impact of type I error inflation could be taken into account. Causality also cannot be assumed because there is no possibility to determine the directionality of the relations presented with this type of methodological design. However, cross-sectional studies can be useful for public health planning [46] and to guide preventive programs.

There are other variables that exert influence on drug use by adolescents (e.g. peer and parental influence) and that were not controlled for in this manuscript. Regarding the motivation variable, we speculate that separating health reasons from keeping fit could have some impact on results. These motivations involve different ways to face the practice of sport. Health reasons are often linked to a regular practice aiming at better general body functioning, while the practice oriented to keeping fit is more associated to aesthetics. However, we were not able to identify such differences. Future studies should be aware of this variable. Beyond that, this study did not differentiate exercise from physical activity. Physical activity corresponds to any body movement that results in energy expenditure whereas exercise is a repetitive and planned activity aiming a health improvement [47]. In our case, exercise was not specifically measured.

## Conclusions

Sports are usually related to healthy habits in several contexts, but this study concludes that this assumption cannot be adopted for the relationship between soccer and heavy episodic drinking among Brazilian adolescents. The association between soccer and HED seems stronger than in other team sports. Our study highlighted that the practice of a team sport and its socialization aspects are not the only reason for more HED report among adolescents. Cultural influence could play an important role in alcohol consumption behavior among adolescents, especially in Brazil, where soccer is a highly important sociocultural determinant. Thus, the type of sport and its cultural meaning in the population must be taken into account. Other fundamental variables such as frequency and motivation for practice should also be considered. These data should be integrated into future studies and into prevention or intervention programs for alcohol use that aim for a better approach with students.

## Abbreviations

HED: Heavy episodic drinking; ESPAD: European school survey project on alcohol and other drugs; OR: Odds ratios; aOR: Adjusted odds ratios; 95% CI: 95% of confidence interval

## Competing interests

All authors declare no financial, personal or other competing interests.

## Authors’ contributions

AB conducted the statistical analyses and wrote the first draft of the article as first author; ESO reviewed the statistical analyses. ARN supervised all the steps. All authors interpreted the data, revised critically the manuscript and approved the final version.

## Pre-publication history

The pre-publication history for this paper can be accessed here:

http://www.biomedcentral.com/1471-2458/13/247/prepub
